# Material Properties of Human Bone-Derived Gelatin

**DOI:** 10.3390/polym18141755

**Published:** 2026-07-17

**Authors:** Nikolay A. Ryabov, Larisa T. Volova, Olga A. Karyakina, Sergei S. Ivanov, Violetta V. Boltovskaya, Denis G. Alekseev, Artem D. Volov

**Affiliations:** BioTech Research Institute, Samara State Medical University, Samara 443079, Russia; n.a.rjabov@samsmu.ru (N.A.R.); volovalt@yandex.ru (L.T.V.); o.a.karyakina@samsmu.ru (O.A.K.); koditek00@yandex.ru (S.S.I.); v.v.boltovskaya@samsmu.ru (V.V.B.); a.d.volov@samsmu.ru (A.D.V.)

**Keywords:** allogeneic gelatin, human bone tissue, tissue engineering, spheroids, chondroblasts, 3D bioprinting, biocompatibility

## Abstract

A comprehensive evaluation of allogeneic human bone-derived gelatin (hBG) was conducted as a promising material for regenerative medicine and 3D bioprinting. Comparative analysis with a commercial animal-derived gelatin product (hereinafter CDH) revealed a similar FTIR spectral profile, confirming the retention of key functional groups. Rheological analysis under steady shear showed that hBG is a pseudoplastic fluid with a concentration-dependent viscosity. However, a complete characterization of bioinks requires oscillatory measurements (G′, G″, and gelation point), which are planned in future studies. In vitro experiments on spheroid models (chondroblasts) demonstrated biocompatibility, an absence of cytotoxicity, and support for cell viability in 5% and 10% (hBG-5/10) matrices. Owing to its origin, the material allows for the modeling of “human-in-human” conditions, which makes it a promising precursor for the development of bioinks and matrices in regenerative medicine and tissue engineering. However, definitive positioning of the material as a bioink requires additional oscillatory rheological studies (determination of G′, G″, thixotropy, and gelation point), which were not performed in the present work.

## 1. Introduction

Regenerative medicine and tissue engineering are considered promising fields for restoring damaged tissues and organs, especially in the context of donor material shortages [1,2]. A key technology in this area is 3D bioprinting, which enables the creation of complex tissue constructs with precise architecture mimicking the native structure of tissues [3]. The success of bioprinting is directly dependent on the properties of the bioinks used—materials that must not only provide mechanical support (scaffolding) but also create an optimal microenvironment for cell adhesion, proliferation, and differentiation [4].

Currently, there is a need for biomaterials possessing an ideal balance between physicochemical properties and biological activity. While offering excellent mechanical characteristics, synthetic polymers (such as PCL or PLA) exhibit lower biocompatibility [5]. Conversely, naturally derived materials of animal origin, including gelatin and collagen, are widely used in medicine due to their biomimetic properties; however, their application is associated with several limitations. These include the risk of zoonotic disease transmission (e.g., bovine spongiform encephalopathy), high immunogenicity due to interspecies differences in amino acid sequences, and ethical and religious restrictions [6,7]. In this context, the development of xeno-free materials is becoming a critically important task for the clinical translation of bioprinting technologies [8].

Gelatin is a product of the partial hydrolysis of collagen, the main protein of the extracellular matrix of connective tissue. Owing to its unique structure, which contains RGD (arginine–glycine–aspartic acid) peptide sequences that promote cell adhesion and matrix metalloproteinase (MMP)-sensitive sites for biodegradation, gelatin is one of the most promising biomaterials [9,10]. It possesses thermoreversible gelation properties and can be readily chemically modified (e.g., methacrylation) for photopolymerization, making it an ideal candidate for bioink development [11,12,13].

The choice of bone tissue as the source material for gelatin extraction is not accidental. The bone matrix is predominantly composed of type I collagen (>90% of the organic phase), the main structural protein of bone, which, as part of the osteochondral interface, is also present in the zone adjacent to cartilage. Moreover, bone tissue contains a unique set of non-collagenous proteins (osteocalcin, osteopontin, and bone sialoprotein, as well as a number of growth factors such as BMP and TGF-β) that may be retained in the extracted gelatin in trace amounts. Even partially purified bone-derived gelatin may carry trace levels of these bioactive molecules, potentially providing osteoinductive cues and enhancing osteogenic differentiation of progenitor cells. In contrast, commercial gelatins derived from porcine or bovine skin/hides lack these bone-specific biochemical features. Thus, human bone-derived gelatin (hBG) may serve not only as a xeno-free matrix but also as a microenvironment enriched with molecules favorable for the regeneration of bone and osteochondral defects [14].

The widespread use of commercial animal-derived gelatins (bovine and porcine) in tissue engineering is associated with several immunological risks that are often underestimated in preclinical studies. The most significant of these is the presence of the α-Gal epitope (galactose-α-1,3-galactose), a xenoantigen that causes IgE-mediated allergic reactions in sensitized (alpha-gal syndrome) patients. Recent studies have shown that in patients with alpha-gal sensitization, the use of gelatin-containing hemostatic agents and bioprostheses can lead to delayed urticaria, anaphylaxis, and accelerated implant degeneration. Furthermore, xenogeneic materials carry a risk of transmitting zoonotic pathogens (prions and viruses) and may contain residual endotoxins, even after multi-step purification. In this context, the use of allogeneic, human-derived gelatin represents a fundamentally safer alternative, as it precludes the potential for xenoimmune responses and interspecies pathogen transfer [15,16].

In regenerative medicine, gelatin has found wide application in creating scaffolds for soft and bone tissues, drug delivery systems, and hemostatic agents [17,18]. In the context of bioprinting, gelatin hydrogels provide the necessary viscosity for extrusion and high post-printing cell viability [19]. However, the vast majority of commercially available gelatin is produced from porcine or bovine tissues. The use of gelatin derived directly from human tissues opens new horizons, as such a material completely eliminates the risk of xenogeneic immune reactions and provides a maximally native biochemical milieu for human cells [20,21]. Despite these obvious advantages, studies dedicated to the extraction and application of human-derived gelatin, especially from bone tissue, remain scarce.

Given the above, there is an urgent need to develop standardized protocols for obtaining gelatin from human biomaterial. The aim of the present study is to obtain and comprehensively characterize gelatin from human bone tissue and to evaluate its suitability as a xeno-free biomaterial for applications in regenerative medicine and 3D bioprinting [22]. The use of bone tissue as a source is justified by its high content of type I collagen and the potential specificity of the resulting gelatin for bone tissue engineering [23,24].

A cell-tissue scaffold produced by the 3D bioprinting method must possess the necessary mechanical strength to withstand pressure during the bioprinting process without structural failure at the post-bioprinting stages. Gelatin is known for its good tensile mechanical strength (approximately 17.51 MPa) [25], which is conferred by the complex of triple helices in its structure. Polymer interactions within gelatin contribute to its robust strength, as gelatin is a protein containing non-ionized polar amino acids that form hydrogen bonds, endowing the hydrogel matrix with high adhesive properties [26]. Hydrophilic properties enhance cell viability and proliferation. Gelatin increases the water absorption capacity of the matrix scaffold, as it contains carboxyl and amino functional groups which are also polar and hydrophilic. Such properties are important for stimulating the maturation of gelatin-based cell-tissue constructs. In this context, gelatin hydrogel components act as scaffolds analogous to the extracellular matrix, supporting cell attachment, migration, and differentiation. Following regeneration, the gelatin hydrogel is replaced by the body’s own natural cells and tissues.

Based on the analysis of the relevant scientific literature, the present work represents the first comprehensive study of gelatin derived from human bone tissue, which has been pretreated using a previously patented method (RU2366173C1). The novelty of this research lies in three aspects:

1. A detailed physicochemical characterization of hBG was performed, including molecular weight distribution by SDS-PAGE, ash content analysis, and rheological behavior over a wide range of temperatures and shear rates.
2. The biocompatibility of this material was evaluated using 3D human chondroblast spheroids in two configurations: on the material surface and upon encapsulation.
3. The rheological profile of hBG under steady shear is presented, which is a necessary (though not sufficient) step towards assessing its potential suitability as a bioink component. The rheological profile of hBG under steady shear is presented. These data are preliminary and allow only a qualitative assessment of the pseudoplastic behavior of the material. A definitive conclusion on the suitability of hBG as a bioink component requires oscillatory measurements (G′, G″, thixotropy, and gelation point), which are planned for the subsequent stages of the study.

The experimental part of the work involved the following objectives:

1. To obtain gelatin extract from human bone tissue.
2. To determine the mass fraction of protein and product yield in the obtained gelatin.
3. To characterize the chemical structure and functional groups of the gelatin using FTIR and ultraviolet (UV) spectroscopy.
4. To determine the molecular weight profile of the gelatin by sodium dodecyl sulfate-polyacrylamide gel electrophoresis (SDS-PAGE).
5. To investigate the rheological properties of gelatin solutions as a function of concentration and temperature in comparison with a known commercial product.
6. To determine the mechanical properties (Young’s modulus) of the formed gelatin hydrogels.
7. To generate spheroids from primary human chondroblasts and assess their viability using culture, morphological methods, and fluorescent vital dyes.
8. To conduct a comparative analysis of spheroid viability both in the presence of and upon encapsulation in allogeneic human bone-derived gelatin versus a commercial gelatin (BioloT LLC, Russia).

## 2. Materials and Methods

### 2.1. Gelatin Preparation

**Figure 1:**
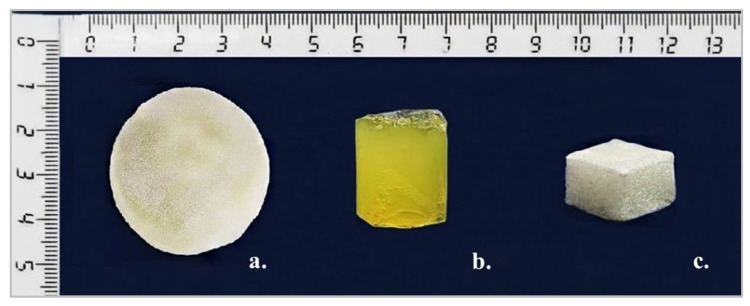
Study objects: (**a**) cancellous tissue of the femoral head (cross-section); (**b**) human bone-derived gelatin (hBG) solution; (**c**) lyophilized gelatin gel.

This section presents the developed process for obtaining gelatin gels from the supporting tissues of human cadaveric material, specifically resected femoral heads (Figure 1a). The study protocol was approved by the Local Ethics Committee of Samara State Medical University (Protocol No. 239 dated 10 November 2021). All stages and operations comprising the proposed technological scheme were carried out in accordance with ISO 13485:2016 “Medical devices—Quality management systems—Requirements for regulatory purposes” [27].

Cadaveric bone tissue, obtained from screened donors (with laboratory confirmation of the absence of hemotransmissible infections), was processed according to a previously patented method (RU2366173C1). The processing included stages of mechanical cleaning, demineralization, and degreasing. Subsequently, gelatin extraction from the obtained biomaterial was performed using deionized water. For this purpose, the allogeneic biomaterial, prepared and purified in the preceding stages, was frozen and ground in an “IKA A11 basic” laboratory mill (IKA^®^-Werke, Staufen, Germany) until a homogeneous mixture was obtained. Extraction was then carried out initially at a temperature of 60–80 °C for 4 h, followed by 90–100 °C for 7 h, maintaining a ratio of 1:6 of the extracted material (60 g of bone tissue) to the extractant (360 mL of deionized water) and an acidic pH value (3.0–5.5). The resulting extract was subsequently filtered sequentially through paper filters until a clear liquid free of mechanical inclusions was obtained. The gelatin solution was then concentrated, followed by neutralization to a pH of 7.0–7.4, monitored using a “Hanna HI 8314” liquid pH meter–analyzer (Hanna Instruments, Vöhringen, Germany). The resulting neutral solution was purified by dialysis against deionized water for 48 h, with the dialysate changed every 12–16 h. The purified extract from the allogeneic biomaterial was left for 2 h at a temperature of +4–7 °C, yielding a gelatin gel with a dense and viscous structure (Figure 1b).

Lyophilization of the gelatin gel was performed using an “AK 2-55” freeze-dryer (LyoGene, St. Petersburg, Russia). The process duration was 18 h. The condenser temperature was maintained at −55 °C. Vacuum was applied stepwise: In the first stage (during the first 6 h), the chamber pressure was 3.00 mBar; in the second stage (subsequent 12 h), the pressure was reduced to 0.15 mBar. The shelf temperature of the freeze dryer was increased to +60 °C during the process.

The gelatin gel lyophilisate is a porous sponge, white to cream in color (Figure 1c), free of mechanical inclusions, odorless, and stable during storage in sealed packaging while maintaining a temperature regime of (20 ± 5) °C. The quality of the finished gelatin hydrogel lyophilisate was ensured through strict adherence to technological procedures performed on the equipment.

The lyophilisate was placed in sealed glass vials or vacuum-sealed bags and then into standard sterilization packaging. The final product was subjected to gamma sterilization. Testing for microbiological purity was conducted in an accredited microbiology laboratory in accordance with ISO 11737-1:2018 “Sterilization of health care products—Microbiological methods. Part 1: Determination of a population of microorganisms on products” and ISO 11737-2:2019 “Sterilization of health care products—Microbiological methods. Part 2: Tests of sterility performed in the definition, validation and maintenance of a sterilization process” [28,29].

During the study, three batches of the lyophilized form of gelatin were prepared, from which samples of allogeneic human bone-derived gelatin (human bone gelatin) with concentrations of 5%, 10%, and 15% (hereinafter referred to as hBG-5, hBG-10, and hBG-15, respectively) were obtained.

As the studied gelatin is non-crosslinked and water-soluble, assessment of enzymatic degradation with collagenase was not performed due to the inability to separate the contributions of dissolution and enzymatic hydrolysis.

### 2.2. Quantitative Assessment of the Gelatin Sample

#### 2.2.1. Moisture Content

The moisture content of the lyophilized product was determined by the thermogravimetric method, with sample weighing performed on a laboratory analytical balance. For this purpose, a 2 to 5 g portion of the lyophilized gelatin sample was placed in a drying oven and dried at a constant temperature of (105 ± 2) °C for two hours to constant mass. The mass fraction of moisture was calculated using the formula:

(1) $$X,\;\%\;=\;\frac{\left(m-m1\right)\times 100}{m},$$

where *m*—mass of test sample before drying, g; *m*1—mass of test sample after drying, g; 100—conversion factor to percentage.

#### 2.2.2. Mass Fraction of Total Nitrogen

The total nitrogen content in the obtained lyophilized sample was determined by the Kjeldahl method [30], which is based on the mineralization of the test material with concentrated sulfuric acid using a catalyst (copper sulfate) to convert organic nitrogen into ammonium ions, alkalization of the resulting solution, distillation of the released ammonia into an excess boric acid solution, titration with hydrochloric acid to determine the amount of ammonia bound by the boric acid, and calculation of the mass fraction of nitrogen in the sample based on the amount of ammonia formed. A “PMP-8M” digester (VILITEK LLC, Moscow, Russia) was used for sample mineralization. Ammonium sulfate was converted to ammonia, which was distilled on an “AKV-10” semi-automatic Kjeldahl distillation unit (VILITEK LLC, Moscow, Russia) with steam into a boric acid solution. The mass fraction of ammonia was then determined by the titrimetric method using a manual titration setup.

The mass fraction of nitrogen was calculated using the formula:

(2) $$X,\;\%\;=\;\frac{0.0014\times \left(V1-V2\right)\times 100\times K}{m}$$

where 0.0014—amount of nitrogen equivalent to 1 cm^3^ of 0.1 mol/dm^3^ acid solution; *V*1—volume of 0.1 mol/dm^3^ hydrochloric acid solution used for titration of the sample, cm^3^; *V*2—volume of 0.1 mol/dm^3^ hydrochloric acid solution used for titration in the blank control measurement, cm^3^; *K*—correction factor for the concentration of the 0.1 mol/dm^3^ hydrochloric acid solution; *m*—mass of the sample, g.

#### 2.2.3. Mass Concentration of Total Protein

The mass concentration of total protein was determined by the colorimetric method using biuret reagent. The method is based on the interaction of divalent copper ions with peptide bonds of the protein molecule in an alkaline medium, resulting in the formation of a colored complex.

For this purpose, a gelatin solution with a concentration of (60–80) mg/mL was prepared. Then, 5 mL of biuret reagent was added to 0.1 mL of the test solution, and the optical density was measured at a wavelength of 540 nm. The total protein concentration was calculated using a calibration curve. Bovine serum albumin (calibrator 70 g/L, “Total Protein-Olvex” kit, Catalog No. 006.001) at various dilutions was used as a standard for constructing the calibration curve.

#### 2.2.4. Quantitative Analysis of Material Purity—Residual Mineral Content (Ash Content)

Quantitative determination of residual ash in the obtained gelatin was performed by gravimetric analysis after incineration at 600 ± 25 °C in a muffle furnace to constant mass.

### 2.3. FTIR Spectroscopy

Spectra of the lyophilized product were recorded using an FT-801 Fourier-transform spectrometer (SIMEX LLC, Moscow, Russia) equipped with an attenuated total reflectance (ATR) attachment under the following parameter values:

- Spectral resolution: 8 cm^−1^;
- Number of scans (accumulations) for spectrum acquisition: 100.

No preliminary sample preparation was performed. Commercial gelatin “CAS-9000-70-8” (CDH, New Delhi, India) was used as a reference sample.

### 2.4. UV Spectroscopy

Intrinsic absorption spectra were recorded on an “SM 2203” spectrofluorometer (SOLAR, Minsk, Belarus) in the 220–600 nm range, with a 10 mm path length. An aqueous solution of the material with a mass concentration of nitrogenous substances of approximately 2 mg/mL was prepared beforehand. Commercial gelatin “CAS-9000-70-8” (CDH, India) was used as a reference sample.

### 2.5. Assessment of Structural-Mechanical and Rheological Properties

The rheological characteristics of the samples were determined using an “RM 200 CP4000” rotational rheometer (Lamy Rheology SARL, Champagne-au-Mont-d’Or, France). This instrument was used to measure viscosity as a function of shear rate and temperature. The rheometer was equipped with a corresponding CP6030 measuring spindle for precise control of shear geometry and to ensure data reproducibility.

Determination of the mechanical strength and textural properties of the samples was performed using a “TX-700” texture analyzer (Lamy Rheology SARL, Champagne-au-Mont-d’Or, France). This instrument was used to measure the material’s resistance to deformation, enabling the quantitative assessment of parameters such as hardness or gel strength, using a defined load and deformation rate profile. A CPF attachment with a Pt100 force sensor was used for this purpose.

Young’s modulus was used as an indicator characterizing the elastic properties of the gelatin solution. For this purpose, a compression test was conducted on several samples of gelatin sponge solution on the texture analyzer. The gelation ability of the gelatin samples was assessed by the Bloom method [31]. The studied samples of hBG gelatin sponge solutions were in the form of disks with a diameter of Ø 10 mm and a height of 5–7 mm. To form gels, the solutions were cast into molds and kept at +4 °C for 12 h. All mechanical tests (compression for Young’s modulus determination and Texture Profile Analysis (TPA)) were performed at +4 °C using the cooled platform of the texture analyzer. At this temperature, non-crosslinked gelatin forms a stable physical gel, enabling correct measurement of its elastic properties; at 37 °C the gel melts, as confirmed by the rheological data.

The use of two types of instruments (a rheometer for flow and viscosity analysis, and a texture analyzer for mechanical strength) ensured a comprehensive approach to the evaluation of the physicochemical properties of the systems under investigation.

The mechanical tests in this study were performed on non-crosslinked gelatin gels formed at +4 °C. These tests (Young’s modulus and Bloom strength) serve as a primary quality assessment of the starting gelatin and its physical gelation capacity. Since the ultimate application of the material in bioprinting requires chemical crosslinking (e.g., methacrylation), more complex viscoelastic studies (stress relaxation and mechanical profile modeling) were not performed at this stage—they will be carried out after stabilization of the material, when the mechanical properties become practically relevant for long-term constructs.

An important limitation of the present study is that all rheological measurements were performed under steady shear conditions. Dynamic parameters (storage modulus G′, loss modulus G″, thixotropic recovery, and sol–gel transition temperature) were not determined due to the technical capabilities of the rotational viscometer used. Therefore, the interpretation of the rheological data is restricted to describing the apparent viscosity under shear and does not allow prediction of the material’s behavior during printing and post-printing structuration.

### 2.6. SDS-PAGE Electrophoretic Analysis

Electrophoretic analysis of the lyophilized gelatin samples was performed in 12% polyacrylamide gel (PAAG) according to the Laemmli method (1970) [32].

A “WIX-easy PRO4 Mini” vertical electrophoresis mini-chamber (Wix Technology Co., Ltd., Beijing, China) with 7 × 8 cm glass plates was used. “All Blue Prestained Standards” protein markers (Bio-Rad, Hercules, CA, USA) for a 4–20% gradient gel with molecular weights of 250–10 kDa and three reference proteins at 75, 50, and 25 kDa were employed. A total of 10 µL of the original solution was loaded per lane.

The sample weights were 5.0 mg. The studied protein was solubilized in 0.0625 M Tris-HCl buffer, pH 6.8, containing 4 M urea and 4% sodium dodecyl sulfate. The samples were dissolved in 500 µL of sample buffer. Before analysis, β-mercaptoethanol (5% of the obtained volume; 25 µL) was added. The samples were incubated in a boiling water bath for 5 min. A 0.04% bromophenol blue solution was used as a tracking dye. The prepared samples were loaded onto PAAG tracks (100, 150, and 300 µg).

The commercial CDH gelatin solution was prepared analogously to the hBG samples. In total, 10 µl of this solution were loaded per well, corresponding to 50 µg of total protein. The loading volume was chosen to ensure clear visualization of all major bands without overloading the gel, thus allowing a correct comparison of the hBG and CDH electrophoretic profiles.

Electrophoresis was conducted at a voltage of 120–180 V for 3 h and 40 min. Gels were stained with Coomassie G-250 solution and subsequently washed twice with 10% ethyl alcohol containing 8.5% acetic acid, followed by 7% acetic acid (2–3 days).

For quantitative assessment of the identified fractions, the “Image Lab Version 6.1.0 build 7 Standard Edition” gel documentation system (Bio-Rad Laboratories, Hercules, CA, USA) was used.

### 2.7. Cultural and Morphological Research Methods

The work was carried out in the Department of Cell Technologies at the Institute of Biotechnology “BioTech”, Samara State Medical University. Sample testing was conducted in accordance with ISO 10993-5:2009 Biological evaluation of medical devices. Part 5: Tests for in vitro cytotoxicity [33] and the OECD Principles of Good Laboratory Practice (GLP) [34]. The department’s laboratory is certified, as confirmed by Certificate No. 25.0151.026 dated 10 March 2025 (Russian Register Certification Association).

#### 2.7.1. Equipment Used

We used a Class II biological safety laminar flow hood “BAVp-01 Laminar-S” (Laminar Systems, Miass, Russia); CO_2_ incubator “MCO-18AC” (Sanyo, Osaka, Japan); refrigerated centrifuge “5702R” (Eppendorf, Hamburg, Germany); visualization system—a hardware–software complex (HSC) based on an “Olympus IX 83” inverted microscope (Olympus, Tokyo, Japan) with a fluorescence module, phase contrast, and a “DP74 SC100” color digital CCD camera (Olympus, Tokyo, Japan), and a desktop computer, with “CellSens Dimensions V2” software (Olympus, Tokyo, Japan); fluorescence microscope “Leica DMIL LED” (Leica, Wetzlar, Germany); and automated digital cell counter “Luna II” (Logos Biosystems, Gunpo-si, Republic of Korea).

#### 2.7.2. Reagents and Materials Used

We used disposable plasticware of “cell culture” grade—25 cm^2^ and 75 cm^2^ culture flasks (TPP, Trasadingen, Switzerland); 48-well flat-bottom plates (TPP, Trasadingen, Switzerland); a culture plate for spheroid formation, “Ultra Low Attachment” surface (Sarstedt, Nümbrecht, Germany); reagents for cell cultivation of “cell culture” grade: fetal bovine serum, liquid, sterile; Medium 199 with L-glutamine, liquid, sterile; Versene solution 0.02%, sterile; Trypsin solution 0.25%; Ehrlich’s hematoxylin (BioloT LLC, St. Petersburg, Russia); and 0.1% gelatin (BioloT LLC, Russia). Sudan III dye (BioVitrum LLC, Russia) was also used. For cell visualization, the “LIVE/DEAD TM Cell-Mediated Cytotoxicity Kit” fluorophore kit (Invitrogen, Carlsbad, CA, USA) and trypan blue 0.4% solution (Invitrogen, Carlsbad, CA, USA) were used.

#### 2.7.3. Study Procedure

Spheroids were generated from a primary culture of chondroblasts obtained from fragments of human nasal septum cartilage (Ethical Approval: The study protocol was approved by the Local Ethics Committee of Samara State Medical University, Protocol No. 239 dated 10 November 2021). Cells were cultured using a modified authorial technique for primary explants described in one of our previous studies [35]. Cells were detached from the bottom of the parent culture flask using a standard method. Culture viability was assessed using the fluorophore kit according to the manufacturer’s protocol.

For visualization of live and damaged cells on the surface of the test samples, the LIVE/DEAD™ Cell-Mediated Cytotoxicity Kit (Invitrogen, Carlsbad, CA, USA) was used according to the manufacturer’s protocol. The dyes included in the kit (calcein-AM and ethidium homodimer-1) are synthetic fluorogenic chemical probes that are classified as vital dyes because they do not exert significant cytotoxic effects and allow staining of live cells without fixation. Calcein-AM (a fluorescein derivative) freely crosses the cell membrane and is hydrolyzed by intracellular esterases of viable cells to yield fluorescent calcein, which emits green fluorescence (λex ~490 nm, λem ~515 nm). Ethidium homodimer-1 is an aromatic cationic compound that only penetrates damaged membranes and, upon binding to nuclear DNA, emits red fluorescence (λex ~535 nm, λem ~617 nm). Thus, live cells are visualized in green, while dead cells display a bright red nucleus.

The seeding dose was calculated using the vital dye trypan blue (1000 cells per well of a 96-well plate for spheroid formation). Cells were observed daily using an inverted microscope. On day 5, the formed spheroids were retrieved using sterile 1000 µL pipette tips. Culture medium was added to the tips to prevent spheroid adhesion to the walls. The formed spheroids were carefully collected and transferred to a 5 mL centrifuge tube with a blue cap. The sample was centrifuged at 500 rpm for 5 min. The supernatant was aspirated, leaving the required volume of fluid.

To assess cell viability in spheroids in the presence of experimental gelatin gels derived from human bone tissue at concentrations of 5% and 10%, the study was conducted in two ways:

- Method 1: Seeding spheroids onto the surface of experimental hBG gelatin samples. A total of 200 µL of 5% and 10% gelatin was added to the wells of a 48-well plate, after which a spheroid was seeded onto the surface. The plate was placed in a CO_2_ incubator for 30 min, and then the wells were filled with complete culture medium up to 400 µL.
- Method 2: Embedding spheroids within the gelatin composition. Spheroids were introduced into a syringe containing the loaded gelatin and mixed by transferring back and forth 2–3 times into another syringe using a Luer connector, thereby evenly distributing the spheroids within the sample.

A 0.1% gelatin solution (BioloT LLC, St. Petersburg, Russia) was used as the control material. The procedure for introducing spheroids into the control material was identical to that used for the study groups of gelatin.

The control and test samples were cultured under CO_2_ incubator conditions at constant humidity and temperature. The study duration was 3 days. The condition of the spheroids in both the experimental and control wells was examined daily.

At the end of the study, all samples were stained using fluorescent dyes, as well as morphological staining with Ehrlich’s hematoxylin and Sudan III. A quantitative assessment of the proliferation and growth of cells from spheroids seeded onto the surface of the samples was performed.

The quantitative assessment included calculation of the proliferation index, doubling time, and number of doublings.

The proliferation index was calculated using the formula:

(3) $$\mathrm{IP}=\frac{N_{t}}{N_{0}}$$

where *N_t_* is the number of cells in the monolayer after *t* hours of cultivation; *N*_0_ is the number of cells in the monolayer, taken as the initial value.

Adherent “mortal” cell cultures grow unevenly between passages, as the principle of “contact inhibition” comes into effect with a decrease in free surface area, and the proliferation of the culture as a whole slows down. Considering the above, our laboratory’s practice is to determine the IP every 24 h, with the first *N*_0_ value being the monolayer density 24 h after cell seeding, and *N_t_* taking the form *N*_24_.

The culture doubling time was calculated using the formula:

(4) $$\mathrm{TD}\;=\;t\times \frac{lg\;2}{lg\;\left(N_{t}/N_{0}\right)}$$

where *t* is the culture growth time (hours); *N*_0_ is the initial number of cells; *N_t_* is the number of cells after *t* hours.

Taking into account the aforementioned points regarding the growth of adherent cells, the doubling time should also be calculated every 24 h. In this context, the value of *t* takes the form of 24 (hours).

The number of culture doublings (ND) was assessed over the entire logarithmic phase according to the formula:

(5) $$\mathrm{ND}=\frac{\left(lg\;N_{t}{-lg\;N}_{0}\right)}{lg\;2}$$

where *N*_0_ is the number of cells 24 h after seeding; *N_t_* is the number of cells at the end of the logarithmic phase.

### 2.8. Data Processing

For all quantitative studies, three biological replicates (*n* = 3) were used, where each replicate corresponded to a sample from an independent experiment. Given the small sample size, the application of parametric tests (and any statistical significance tests) was deemed inappropriate. Therefore, statistical processing was limited to methods of descriptive statistics. The results are presented as the arithmetic mean (M) and standard deviation (SD); where appropriate, the median (Me) is additionally indicated. Calculation of *p*-values was not performed, as with *n* = 3, any conclusions regarding statistical significance of differences would be methodologically unsound and could lead to an overestimation of the observed effects.

Given the small sample size (*n* = 3 per group), inferential statistical tests (e.g., *t*-test, ANOVA) were not performed, as any conclusions regarding the statistical significance of differences would be methodologically unsound. The results are presented as the arithmetic mean (M) and standard deviation (SD); the median (Me) is additionally indicated where appropriate. The obtained data should be interpreted as descriptive trends identified in a pilot study.

FTIR and UV spectra and rheological curves are presented as averaged dependencies (from three biological replicates). Statistical processing of the obtained data was performed using the software package “STATISTICA 12.5.192.7” (StatSoft Inc., Tulsa, OK, USA).

## 3. Results and Discussion

During the conducted study, a technological process for obtaining gelatin from human bone tissue was refined. The dry matter product yield averaged 70% across batches.

### 3.1. Quantitative Assessment of Gelatin

The residual moisture content of the lyophilized gelatin solution was approximately 1%, which conforms to generally accepted standards for lyophilized forms of biopreparations (1–5%). It should be noted here that exceeding the standard values for residual moisture reduces product stability.

**Figure 2:**
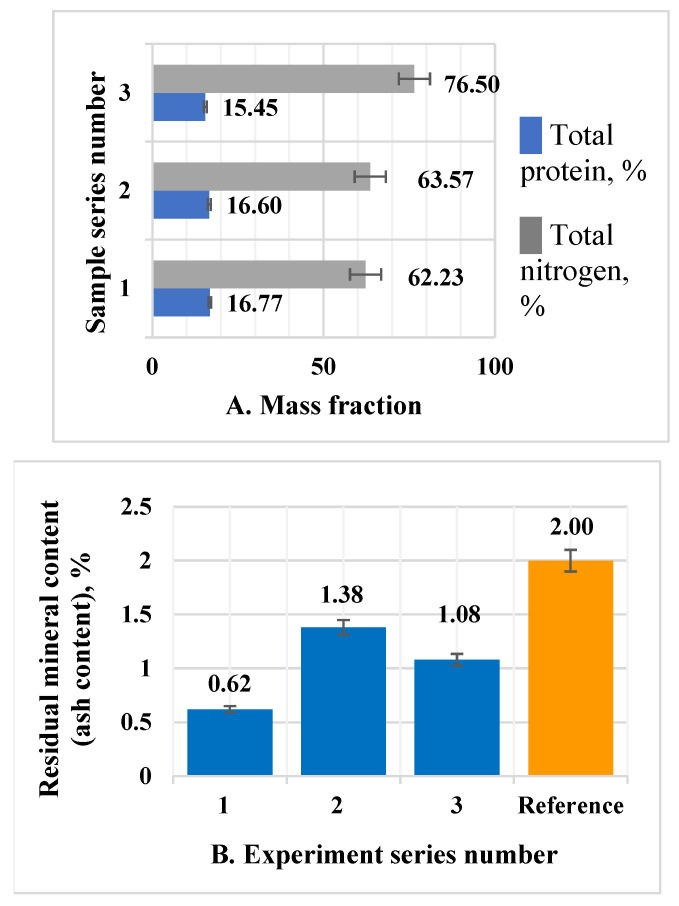
(**A**) Bar chart of the quantitative assessment of protein in gelatin samples from 3 batches; (**B**) residual mineral content (ash content).

The mass fraction of total nitrogen in the obtained product batches ranged from 15.45% to 16.77% (M = 16.27%; SD = 0.72%; Me = 16.60%). The mass fraction of total protein ranged from 62.23% to 76.50% (M = 67.43%; SD = 7.88%; Me = 63.57%) (Figure 2A).

The residual mineral content (ash content) for the obtained gelatin batches ranged from 0.60% to 1.42% (M = 1.03%, SD = 0.38%, Me = 1.08%) (Figure 2B).

The low ash content of the obtained gelatin (less than 2%) indicates a high degree of demineralization and washing efficiency, confirming the purity of the material and the absence of significant mineral impurities. The obtained value complies with pharmacopeial purity standards for preparations, which is an important advantage for biomedical applications (reducing the risk of inflammatory reactions associated with calcium phosphate crystals).

The allogeneic nature of the investigated hBG gelatin deserves special attention. Unlike commercial animal-derived gelatins widely used in tissue engineering and bioprinting, hBG does not contain xenoantigenic determinants, in particular the α-Gal epitope, which is recognized as a key factor in the immunogenicity of xenogeneic biomaterials. Although manufacturers of commercial gelatins employ multi-step purification to remove α-Gal, the complete elimination of this epitope is difficult to achieve, and residual amounts can provoke adverse immune reactions in sensitized patients, as shown in recent studies of decellularized xenogeneic matrices [15,16]. The use of allogeneic hBG gelatin completely eliminates this risk, which is especially important in the context of potential clinical application for articular cartilage regeneration, where chronic immune inflammation could completely negate the therapeutic effect. It should be noted, however, that the allogeneic approach does not eliminate the need for thorough donor screening for infectious agents (HIV, hepatitis, and syphilis) in accordance with regulatory requirements, which is a standard procedure for all human-derived biomaterials.

Although the low ash content and sterility (gamma sterilization) confirm a high degree of purification, endotoxin levels were not quantified in this study. This represents a limitation that will be addressed prior to in vivo experiments.

### 3.2. FTIR Spectroscopy Results

The infrared absorption spectra of allogeneic gelatin, allogeneic demineralized bone tissue, and commercial gelatin exhibit peaks reflecting the structural features of protein compounds in the studied materials. An important aspect is the preservation of the collagen polypeptide chains and the secondary protein structure.

**Figure 3:**
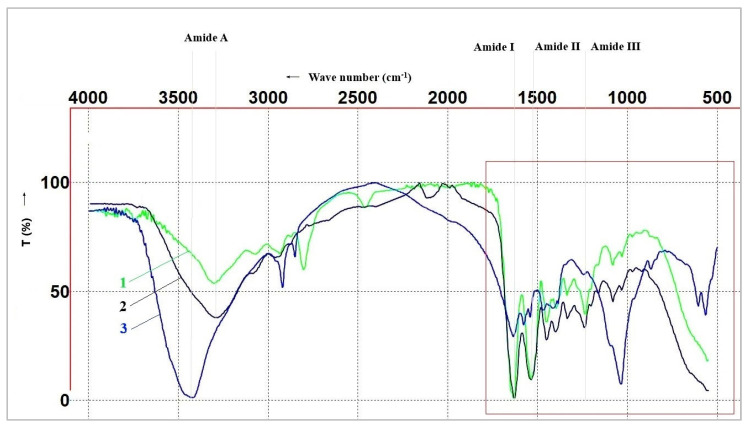
IR absorbance spectra of allogeneic gelatin (1, green line), commercial CDH gelatin (2, black line), and allogeneic bone-derived gelatin hBG (3, blue line).

The broad peak in the region of 3600–3200 cm^−1^ (“Amide A”) is attributed to N-H stretching vibrations of peptide bonds. The position of this peak depends on the involvement of the N-H group in hydrogen bond formation. The peak position at a higher wavenumber in the bone tissue spectrum may be explained by weaker CO···HN-type hydrogen bond interactions due to reduced hydration compared to gelatin and steric constraints within the bone tissue structure [36,37,38] (Figure 3).

The absorption band in the region of 1640–1620 cm^−1^ (“Amide I”) in the hBG and CDH gelatin spectra demonstrates the integrity of the main peptide chain and secondary structure. Absorption bands in this region are caused by C=O and N-H stretching vibrations [37,38] (Figure 3).

The peaks of C-N stretching and N-H bending vibrations (“Amide II”) in the region of 1550–1530 cm^−1^ are directly related to the secondary protein structure (alpha-helix, beta-sheets, triple helix, or random coil). In pure gelatin, the residual mineral component is absent; consequently, a classical peak position around 1540 cm^−1^ is observed, in contrast to the bone tissue spectrum, where a complex pattern and a peak shift to 1577–1585 cm^−1^ are seen in this region. This shift can be explained by the interaction of collagen side-chain amino acid residues with residual calcium ions, or by the influence of non-collagenous bone proteins (osteocalcin, osteonectin, etc.) [39,40] (Figure 3).

Characteristic vibrations of the amide group and bending vibrations of the amino acid carbon skeleton comprising the polypeptide chain of the study material in the region of 1250–1230 cm^−1^ (“Amide III”) also reflect the polypeptide chain structure. The shift of this peak towards higher wavenumbers and the decrease in its intensity may be associated with the preservation of the rigid triple helix structure in bone tissue and its overlap with the residual mineral component of bone tissue [38,39,40,41,42] (Figure 3).

The IR analysis results for the studied gelatin samples are consistent with the literature data and demonstrate differences between demineralized bone collagen, which is characterized by a rigid triple helix structure, and gelatin, which is a denatured collagen in the form of a “random coil,” in which irregular conformations predominate, yet the ability for partial recovery of the secondary protein structure is retained.

### 3.3. UV Spectroscopy Results

**Figure 4:**
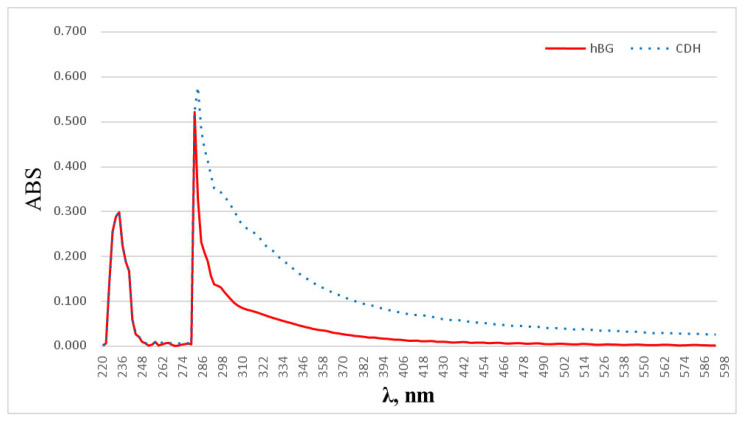
UV intrinsic absorbance spectra of hBG and CDH sample solutions.

The intrinsic UV absorption spectra of gelatin solutions exhibit characteristic maxima at 234 nm and at 282–284 nm [36] (Figure 4). Absorption around 230 nm is attributed to the peptide bonds of the polypeptide backbone, while absorption around 280 nm is due to aromatic amino acids (tyrosine and phenylalanine) [43], which are present in both type I collagen and non-collagenous proteins.

In the hBG samples, an intense peak at 284 nm was recorded. Pure type I collagen contains minor amounts of phenylalanine and tyrosine residues that naturally absorb light in this region. At the same time, bone tissue contains unique non-collagenous proteins (osteocalcin, osteopontin, and bone sialoprotein), which are also rich in aromatic amino acids and may be retained in trace amounts during extraction, contributing to UV absorption. However, since no targeted immunochemical analysis (e.g., ELISA or Western blotting with specific antibodies against osteocalcin or osteopontin) was performed in this study, we cannot unambiguously claim that the observed peak is exclusively due to the presence of these non-collagenous proteins. It may also originate from ordinary denatured collagen fractions.

Thus, the interpretation of the peak at 284 nm remains tentative. To confirm the retention of specific bone proteins in hBG, additional studies using ELISA and Western blotting are planned for the next stage of the work.

### 3.4. Results of Assessment of Structural–Mechanical and Rheological Properties

**Figure 5:**
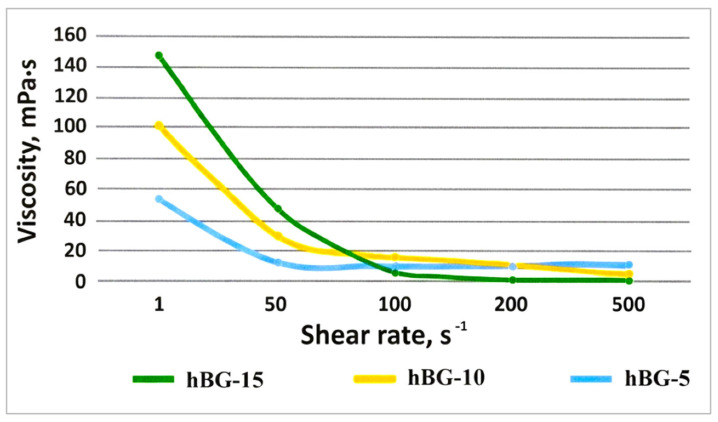
Shear rate sweep plot from rheological measurements of hBG.

Figure 5 presents the results of rheological measurements from a shear rate sweep test performed on the CP-4000 rotational rheometer. This analysis enables the evaluation of viscosity dependence on shear rate for gelatin at different concentrations: hBG-15, hBG-10, and hBG-5.

The abscissa (horizontal axis) represents the shear rate in reciprocal seconds (s^−1^), presented on a logarithmic scale (1, 50, 100, 200, and 500 s^−1^). The ordinate (vertical axis) shows the viscosity in millipascal-seconds (mPa·s).

The graphs clearly demonstrate pseudoplastic behavior for all investigated gelatin concentrations. A decrease in viscosity with increasing shear rate is observed for all samples. The most pronounced drop in viscosity occurs in the low shear rate range (from 1 to 100 s^−1^) [44].

Furthermore, a strong concentration dependence of viscosity is evident: at any given shear rate, the solution viscosity decreases in the following order: hBG-15 > hBG-10 > hBG-5. The hBG-15 gelatin solution exhibits the highest initial viscosity (148 mPa·s at 1 s^−1^), whereas the hBG-5 sample has the lowest viscosity (52 mPa·s at 1 s^−1^), and hBG-10 confirms the concentration-dependent viscosity (102 mPa·s at 1 s^−1^) (Figure 5).

The general trend of increasing viscosity with increasing gelatin concentration is maintained at all investigated shear rates. This underscores the direct dependence of flow resistance on the amount of polymeric material in the system.

**Figure 6:**
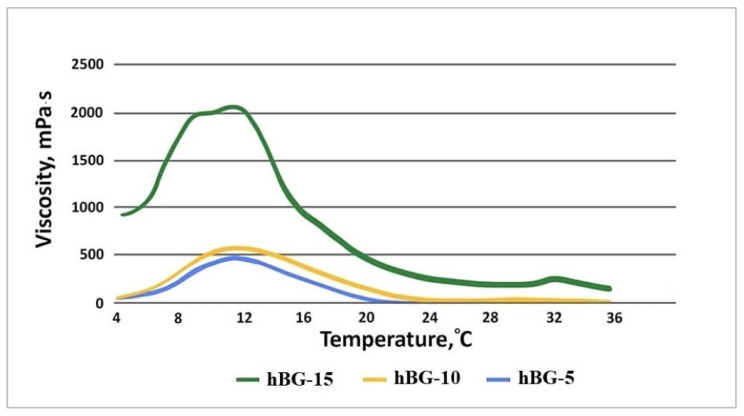
Graph of the temperature dependence of viscosity for hBG samples.

The viscosity curves exhibit a pronounced temperature-dependent character for all investigated gelatin concentrations. On the graph, the horizontal axis represents temperature, varying from 4 to 37 °C. The vertical axis displays viscosity (Figure 6).

At low temperature values of 4–8 °C, all samples (hBG-5, hBG-10, and hBG-15) show relatively low viscosities of 88.3, 99.7, and 973.6 mPa·s, respectively. However, as the temperature increases from 8 to 12 °C, a sharp increase in viscosity is observed for all concentrations, to 463.9, 527.1, and 1995.1 mPa·s, respectively, indicating a significant increase in viscoelastic properties. The temperature sweep was performed in steady-state rotational mode; therefore, the obtained curve reflects apparent viscosity under shear rather than equilibrium gel strength. The temperature ramp was performed in steady rotational mode; therefore, the resulting curve reflects apparent viscosity under shear conditions, not equilibrium gel strength. The relatively low viscosity recorded at 4 °C should not be interpreted as evidence of weak thermodynamic gelation at this temperature. A plausible explanation is that sample loading and the imposed shear partially disrupted the weak physical gelatin network formed during cooling. During subsequent thermal equilibration in the 8–12 °C range, delayed recovery and rearrangement of gelatin chains may have led to a transient increase in apparent viscosity. Above this range, progressive disruption of hydrogen-bonded junction zones and partial melting of gelatin helices resulted in the expected decrease in viscosity. Thus, the maximum observed near 12 °C is interpreted as an apparent rheological peak influenced by measurement history and steady shear, rather than as a true gelation temperature. The hBG-15 solution curve shows the highest spike in viscosity in this range, up to 2074.4 mPa·s (Figure 6).

This is attributed to the fact that as concentration increases, intermolecular interactions, including hydrogen bonds and hydrophobic interactions between segments of polymer chains, are enhanced. This leads to the formation of a more crosslinked and stable three-dimensional network. This network structure offers greater resistance to flow, which manifests as an increase in viscosity [45,46,47].

A decrease in the viscosity of the hBG samples occurs at temperatures of 12–37 °C. After reaching a peak, the viscosity sharply drops for 5%, 10%, and 15% concentrations to 67.1, 162.3, and 371.4 mPa·s at 20 °C, respectively. With a further increase in temperature to 37 °C, the viscosity for 5%, 10%, and 15% concentrations falls to 12.1, 20.3, and 123.5 mPa·s, respectively, corresponding to gel melting and the system’s transition to a more liquid, low-viscosity state. The values provided are for steady-state rotational mode, not oscillatory mode. Therefore, within this experimental setup, it is not possible to correctly determine the storage modulus (G′) and loss modulus (G″), as their calculation requires a mode with periodic perturbing action, which was not used in the measurements on this rheometer.

At higher temperatures, the viscosity of all samples stabilizes at very low values, characteristic of Newtonian or weakly non-Newtonian fluids, where the concentration effect persists, but the decrease in viscosity slows down (Figure 6).

All samples demonstrate the same behavioral pattern upon temperature change, and a dependence of viscosity on concentration is also evident: as concentration increases, viscosity increases.

It should be emphasized that the presented rheological curves were obtained in steady shear mode and reflect apparent viscosity. Comprehensive viscoelastic characterization of hBG, necessary to evaluate its behavior during extrusion and its ability to retain shape after printing, requires oscillatory measurements. Without these data, any claim regarding the suitability of hBG as a bioink is premature. Nevertheless, the observed pseudoplastic behavior and thermosensitivity provide a basis for further investigations in this direction.

**Figure 7:**
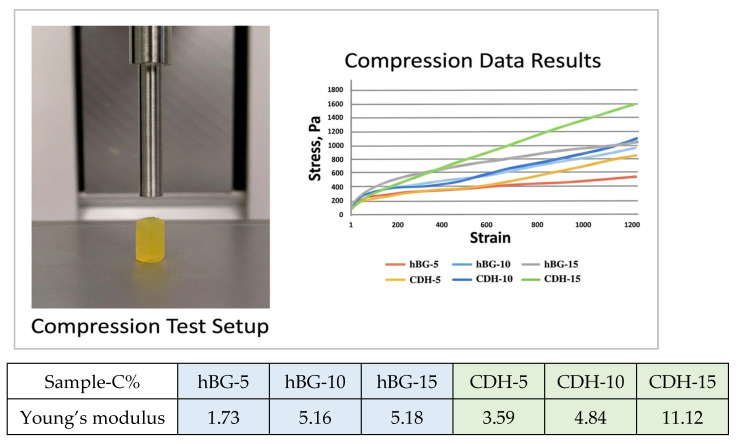
Graph of the study of elastic properties (Young’s modulus) of gelatin sponge solution samples on the TX-700 texture analyzer. Measurements were performed at +4 °C.

When assessing the mechanical properties, the graph in Figure 7 clearly shows how the mechanical resistance of the samples changes depending on their degree of deformation. CDH samples demonstrate noticeably higher resistance to applied stress compared to their allogeneic counterparts (Figure 7). Furthermore, a direct dependence of mechanical properties on concentration is confirmed: the higher the concentration of the investigated component in the system, the higher the achieved values of strength and resistance to deformation.

**Figure 8:**
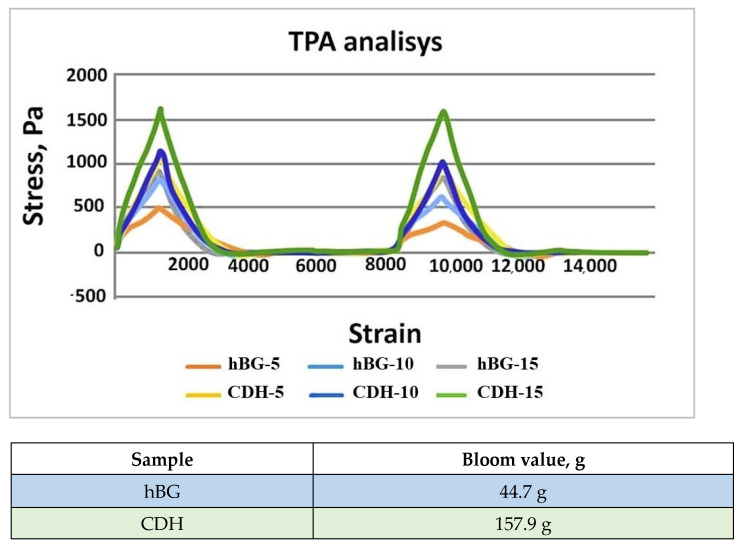
TPA-analysis plot (stress–strain dependence) and Bloom strength values of the commercial (CDH) and developed (hBG) gelatin samples (15% concentration).

The TPA-analysis graph shows the stress–strain dependence for two types of systems—CDH and hBG—at various concentrations. It is evident that under the current conditions, CDH samples demonstrate a significantly higher maximum mechanical resistance of 1579.6 mN compared to allogeneic ones at 955.5 mN at equivalent concentrations of 15%, underscoring their current structural stability (Figure 8).

Moreover, an increase in concentration in both series of samples leads to an increase in strength, which suggests that allogeneic systems could achieve comparable mechanical characteristics with a further increase in their concentration, opening prospects for improving their structure.

For the gelatin samples, an analysis of the Bloom strength value was also conducted. The results of the gelation capacity analysis revealed two qualitatively distinct types of gelatin. The CDH sample, with high gel strength (157.9 g), demonstrates the classical properties of high-molecular-weight gelatin, forming strong and elastic thermoreversible gels.

The hBG sample is characterized by a significantly lower gel strength (44.7 g). The low gel strength of hBG, both in vitro and in vivo, makes it a promising material for creating temporary matrices in tissue engineering, where controlled and rapid resorption is required.

The Young’s modulus of the gelatin gels was determined to be 4.89 kPa from the linear region, highlighted by the blue line (Figure 7).

For all test samples, stress–strain dependencies were constructed, linear segments were identified, and Young’s modulus was calculated. The Young’s modulus values for the gelatin gels are presented in Figure 7. These values range from 1.73 kPa (hBG-5) to 11.12 kPa (CDH-15), indicating a strong dependence of the mechanical response on concentration and origin. The lower Bloom value for hBG indicates that this material forms substantially softer physical gels compared to the investigated commercial CDH gelatin. This property may be promising in a potentially emerging application area where low matrix stiffness and reduced extrusion-associated shear loading are desirable, such as short-term encapsulation, manipulation, or delivery of sensitive cell spheroids. However, this same property limits the shape fidelity after printing and the mechanical stability of non-crosslinked hBG at 37 °C. Its future use in 3D bioprinting should be viewed as an emerging and still-developing direction that requires additional stabilization strategies, such as methacrylation, enzymatic crosslinking, or blending with mechanically reinforcing polymers, followed by systematic rheological, mechanical, and biological validation. The presented rheological data should be considered preliminary in the context of bioink development, since oscillatory measurements (with variation in amplitude, frequency, and temperature) are necessary for a full characterization of viscoelastic properties and the sol–gel transition.

### 3.5. Results of Electrophoretic Analysis of Gelatin Samples (SDS-PAGE)

**Figure 9:**
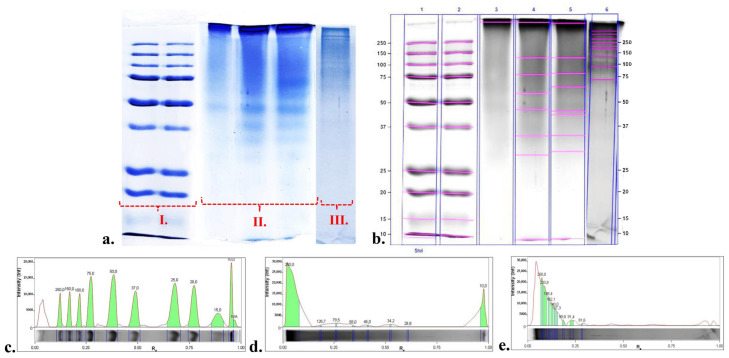
Electropherogram of hBG gelatin proteins (SDS-PAGE, 12% polyacrylamide gel, PAAG): (**a**) electropherogram (general view): I—protein markers; II—hBG gelatin (protein load per track: 100, 150, and 300 µg); III—CDH gelatin standard (protein load 10 µL); (**b**) assessment of identified fractions using the Image Lab Version 6.1.0 build 7 Standard Edition BioRad gel documentation system; (**c**) protein markers; (**d**) hBG gelatin; (**e**) CDH gelatin.

The molecular weight composition of the lyophilized gelatin samples derived from human bone tissue (hBG) was investigated by SDS-PAGE. The analysis showed that the product is a polydisperse system, retaining native type I collagen fractions alongside their hydrolysis products (Figure 9a).

Electrophoretic analysis of the developed gelatin samples was conducted in comparison with a known animal-derived gelatin sample, CDH. The retention of collagen α-chains is critically important for the biological activity of the matrix (Figure 9a,b). A quantitative assessment using the gel documentation system allowed us to establish that the composition of hBG is represented by a polydisperse set of polypeptides with a wide range of molecular weights. Fractions corresponding to collagen α- and β-chains (in the region of 128–79 kDa), as well as low-molecular-weight hydrolysis products (68, 46, 34, and 28 kDa and below), are identified in the sample. The high-molecular-weight region (>250 kDa) is characterized by a weak “smeared” signal, which may indicate the presence of aggregated collagen forms or incompletely denatured fragments. The distinct dominance of discrete fractions with a molecular weight >250 kDa was not detected. The minor component is a reduced form of a 48.8 kDa protein (Figure 9b,d).

The electrophoretic analysis of the CDH gelatin standard indicates a heterogeneous composition and reveals a series of protein bands with continuously decreasing molecular weight. Fractions of 233.9, 189.4, 162.1, 140.0, 127.3, 99.8, 91.4, and 81.6 kDa are identified. While the first four fractions can be correlated with a collagen complex exceeding 250 kDa and aggregated protein chains of lower molecular weight, all subsequent mass values fall within the known characteristics of collagen in the 80–120 kDa monomeric form (Figure 9b,e).

Thus, in all studied gelatin samples, aggregated macromolecular complexes of collagen are retained. Simultaneously, this may be accompanied by the appearance of collagen degradation products of lower molecular weight (Figure 9).

Depending on the specific conditions of the experimental protocol, samples containing collagen and products of its subsequent degradation can be obtained, which is a characteristic of materials produced within the research programs of the Institute of Biotechnology “BioTech” at Samara State Medical University. In general, such products can serve as an optimal matrix for cell culture cultivation, as well as for developing promising wound dressings.

The protein distribution profile in hBG samples demonstrates characteristic differences from collagen hydrolysates. The presence of distinct high-molecular-weight bands (>128 kDa) confirms the material’s high potential for forming a three-dimensional gel network (which correlates with the Bloom strength value).

The nature of the low-molecular-weight fraction is of particular interest. Unlike the classic peptide “smear” resulting from random acid or alkaline hydrolysis, discrete bands (peptides) were identified in the hBG samples. This indicates a specific fragmentation pattern of human collagen macromolecules under the chosen extraction conditions.

The studied hBG gelatin can be characterized as a high-molecular-weight biopolymer with a developed fraction of bioactive polypeptides. The balance between the retained native structures (α- and β-components) and the presence of low-molecular-weight fragments suggests a combination of good gelling properties with high biological activity (assimilability and cell adhesion), making this material promising for application as a scaffold in tissue engineering. Thus, we observe a polydisperse system with the retention of native α- and β-structure and a characteristic fragmentation profile of human bone collagen.

### 3.6. Results of Culture and Morphological Methods

#### 3.6.1. Control Chondroblast Culture

**Figure 10:**
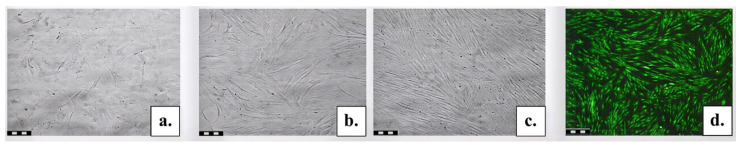
Human chondroblast culture on plastic: (**a**) 1 day after passaging; (**b**) 3 days after passaging; (**c**) 5 days after passaging. Inverted microscope. Phase contrast. Magnification ×100; (**d**) 5 days after passaging; stained with live–dead fluorescent dyes. Fluorescence microscope. Magnification × 100. The scale division in micrographs corresponds to 100 micrometers (µm).

Native culture chondroblasts predominantly had an elongated shape and 3–5 processes. The processes branched out, establishing connections with neighboring cells. The cytoplasm contained many vacuoles in the peripheral zone of the cell. Cell borders were distinct. The nucleus was oval-shaped, typically located centrally, and contained 1–3 nucleoli. The cells exhibited good adhesion to the culture plastic: 2 h after passaging, the majority of chondroblasts attached to the bottom of the culture vessel and spread out. After 24 h, an overwhelming number of cells had attached to the bottom of the culture flask and acquired an elongated form with extended processes (Figure 10a). On day 3, the number of cells gradually increased in the field of view, and they contacted each other via processes (Figure 10b). On day 5, the cells formed a confluent monolayer (Figure 10c). When staining the cells with fluorescent dyes, an even monolayer was observed, and no artifacts were present. Viable cells exhibited uniform green cytoplasmic fluorescence, while damaged cells with bright red nuclei were absent, indicating good cell quality (Figure 10d).

#### 3.6.2. Morphological Study of the Obtained Spheroids

**Figure 11:**
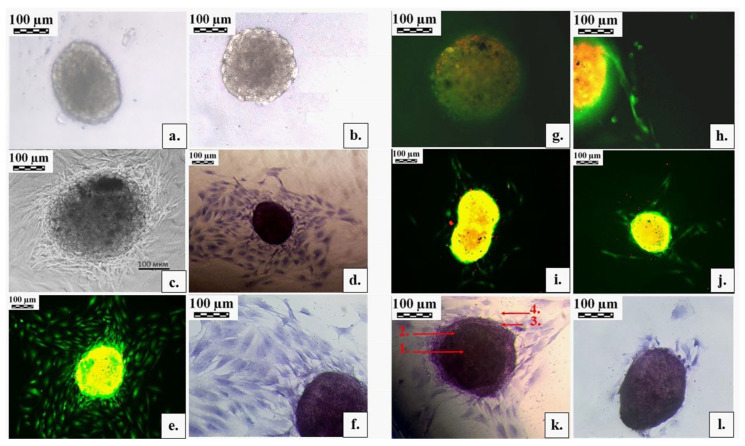
Comparison group: (**a**) general view of a spheroid (24 h post-formation from human chondroblasts); (**b**) dimensions of the spheroid (in µm); (**c**) 3 days after transfer of the spheroid onto the surface of 0.1% gelatin (BioloT LLC, Russia). Native culture. Inverted microscope. Phase contrast. Magnification ×100. (**d**) 3 days after placing the spheroid on the surface of 0.1% gelatin. Stained with Sudan III and Ehrlich’s hematoxylin. (**e**) 3 days after placing the spheroid on the surface of 0.1% gelatin (BioloT LLC, Russia). Stained with fluorescent dyes. LIVE/DEAD TM Cell-Mediated Cytotoxicity Kit. Inverted microscope. Fluorescence module. Magnification ×100. (**f**) Spheroid on the surface of 0.1% gelatin (BioloT LLC, Russia). Stained with Sudan III and Ehrlich’s hematoxylin. Inverted microscope. Magnification ×200. Experimental gelatin samples (3 days from the start of the experiment). (**g**) Spheroid embedded within hBG-10; (**h**) spheroid embedded within hBG-5. Inverted microscope. Fluorescence module. Magnification ×100. (**i**) Spheroid seeded onto the surface of hBG-10; (**j**) spheroid seeded onto the surface of hBG-5. Inverted microscope. Fluorescence module. Magnification ×100. (**k**) Spheroid seeded onto the surface of hBG-10. Spheroid structure: 1—spheroid core; 2—intermediate zone; 3—outer zone; 4—cells emerging from the spheroid. (**l**) Spheroid seeded onto the surface of hBG-5. Stained with Sudan III and Ehrlich’s hematoxylin. Inverted microscope. Magnification ×200.

Samples hBG-5 and hBG-10 were studied using the obtained spheroids. Observation of the plate with formed spheroids after 24 h showed that in all wells, spheroids were nearly uniform in size, spherical in shape, and maintained their shape throughout the experiment (Figure 11a,b).

#### 3.6.3. Study of Spheroids on the Surface of 0.1% Gelatin (BioloT LLC, Russia), Used as a Control Material

A total of 24 h after placing the spheroid on the 0.1% gelatin surface, single cells were observed migrating from the spheroid surface. Their number gradually increased and, by day 3, cells had formed a confluent monolayer at the periphery beyond the spheroid boundaries. Cells connected to each other via 2–3 processes. Borders were clearly defined, the cytoplasm was homogeneous, and a nucleus with 2–3 nucleoli was visualized. The morphofunctional characteristics of the cells were preserved (Figure 11c).

Upon staining with Sudan III and Ehrlich’s hematoxylin, the spheroid was clearly visualized. The central zone was dark, consisting of cellular debris and non-viable cells. Around the spheroid, cells formed a uniform monolayer; cells were in close proximity to each other, connected by 2–3 processes. The cytoplasm was homogeneous, and a nucleus with 2–3 nucleoli was visualized (Figure 11d). Fluorescent staining revealed an increased background fluorescence in the central part of the spheroid, with a narrow green band (viable cells) visible at the spheroid periphery. In the formed monolayer, viable cells were clearly identified. The cytoplasm exhibited homogeneous green fluorescence. Damaged cells with bright red nuclei were absent. This result confirmed the viability of cells within the obtained spheroids (Figure 11e).

#### 3.6.4. Study of Spheroids Embedded Within Experimental hBG Gelatin Samples

Study of the spheroid placed within hBG-10 demonstrated an absence of distinct internal zones. In the central part, background fluorescence was absent; uneven green fluorescence was observed, with areas of pale red staining. In the peripheral zone of the spheroid, a bright green band with isolated red elements was visualized (Figure 11g).

Study of the spheroid in hBG-5 showed a center with enhanced background fluorescence. A narrow green band was visualized in the peripheral zone, indicating the presence of viable cells migrating from the spheroid surface (Figure 11h).

#### 3.6.5. Study of Spheroids Seeded onto the Surface of hBG Gelatin Samples

Examination of the spheroid 3 days after placement on the surface of hBG-10 revealed background fluorescence in the central region of the spheroid. A narrow green band, indicating the presence of viable cells, was observed at the periphery. Cells were noted arranged around the spheroid. The morphofunctional properties of the cells were preserved. The shape was consistent with chondral cells. The cytoplasm was homogeneously stained green; isolated damaged cells with bright red nuclei were present (Figure 11i).

Upon staining the spheroid with Sudan III and Ehrlich’s hematoxylin on the hBG-10 surface, its structure was clearly visualized. The dark-colored central zone comprised cellular debris and non-viable cells. Peripherally, a lighter zone composed of viable cells but with reduced metabolic activity was visualized. The outer zone of the spheroid consisted of actively proliferating cells, as this zone has direct access to oxygen and nutrients from the surrounding culture medium (Figure 11k).

Spheroid on the hBG-5 surface (Figure 11j): Upon examination, the spheroid structure differed somewhat from the previous one. The central part was lighter, with no distinct boundaries between zones. Actively proliferating cells were noted in the outer zone. The morphofunctional state of the cells was preserved (Figure 11).

#### 3.6.6. Quantitative Assessment of Cell Proliferation and Growth

Analysis of the proliferation index, doubling time, and number of doublings revealed the following patterns: On the control material (0.1% gelatin), a slowdown in proliferation was observed by day 3 in contrast to the 5% and 10% gelatin. The proliferation index in the hBG-5 and hBG-10 groups ranged from 1.9 to 2.1, and the doubling time ranged from 22.4 to 25.9 h. These values were comparable to or slightly higher than those in the control group (0.1% gelatin), suggesting that hBG does not impair cell proliferation under the tested conditions. However, due to the limited sample size (*n* = 3), no statistical comparisons were performed, and the obtained results should be considered preliminary.

**Figure 12:**
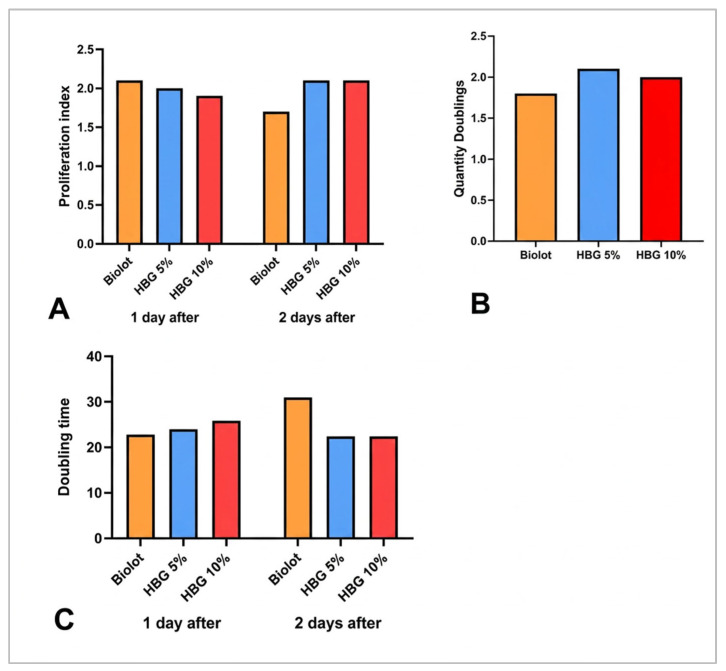
(**A**) Proliferation index; (**B**) number of doublings; (**C**) culture doubling time.

The number of cell doublings over 3 days in the experimental groups (2.0–2.1) was practically no different from the control value (1.8) and even slightly exceeded it (Table 1, Figure 12).

The obtained data show that on the control (0.1% gelatin), the proliferative profile exhibits characteristic dynamics: high activity on day 2 (index 2.1, doubling time ~23 h) is followed by a significant slowdown on day 3 (index decreases to 1.7, doubling time increases to 31 h). This is likely associated with the rapid formation of a confluent monolayer on the liquid substrate, which triggers contact inhibition of growth.

In contrast, on the surface of 5% and 10% gelatin, proliferation remains stably high throughout the observation period. The proliferation index on day 3 not only does not decrease but even slightly increases (up to 2.1), and the doubling time shortens to 22.4 h, corresponding to active logarithmic growth. The number of cell doublings over 3 days in these groups (2.0–2.1) was not lower but even higher than in the control group (1.8). This fact is fundamentally important, as it refutes the potential concern that a higher gelatin concentration (and, consequently, greater gel stiffness and density) might inhibit proliferation.

The absence of significant differences between 5% and 10% gelatin for all parameters studied indicates that within the range of concentrations suitable for bioprinting, the proliferative activity of spheroids does not depend on the exact concentration value. Both options ensure stable cell growth.

Thus, from the perspective of proliferative potential, the use of concentrated gelatin solutions (5–10%) in bioprinting is no less effective than that of a standard culture substrate (0.1%). Moreover, a dense gel can create conditions that prevent premature contact inhibition, making it preferable for the long-term cultivation of spheroids in three-dimensional constructs.

Thus, the conducted studies of the developed hBG samples using in vitro testing on human chondroblast spheroids, with both surface seeding and encapsulation within the gel, confirmed the absence of hBG cytotoxicity, while noting the preservation of cell viability, morphology, and proliferative capacity. The studied gelatin samples preserve cell viability and proliferative capacity, which is a prerequisite for the potential deposition of extracellular matrix. However, direct evidence (e.g., by immunohistochemistry for type II collagen or aggrecan) requires further investigation.

It should be noted that the control material (0.1% gelatin) differs in concentration from the experimental groups (5% and 10% hBG). However, at 37 °C, both hBG-5 and hBG-10 exhibit low viscosities (12.1 and 20.3 mPa·s, respectively), comparable to the viscosity of liquid culture media. Thus, the observed proliferative activity is unlikely to be related to substantial differences in matrix stiffness. Nevertheless, future studies should employ commercial gelatin at corresponding concentrations (5–10%) to rule out a source-specific effect.

## 4. Findings

The research conducted allowed us to draw the following findings:

1. A method for obtaining gelatin from human bone tissue (hBG) was developed and standardized. The lyophilized product is characterized by a residual moisture content of about 1%, which meets the stability requirements for biopreparations. The mass fraction of total nitrogen (15.45–16.77%), total protein content (62.23–76.5%), and low residual mineral content (ash < 2%) confirm a high degree of purification of the protein component.
2. The retention of the secondary protein structure and polypeptide chains was confirmed by FTIR and UV spectroscopy. The spectral profile of hBG demonstrates characteristic bands (Amide A, I, II, and III), indicating the preservation of the collagen polypeptide chain, capable of partial refolding, as well as the presence of non-collagenous proteins characteristic of the bone matrix, distinguishing the obtained sample from commercial analogs.
3. The SDS-PAGE electrophoretic profile of hBG is characterized by polydispersity with major protein bands in the range of 128–28 kDa, corresponding to collagen α- and β-chains and products of their partial hydrolysis. High-molecular-weight aggregates (>250 kDa) are present as a minor fraction.
4. hBG gelatin solutions exhibit pseudoplastic behavior (viscosity decreases with increasing shear rate) and possess notable thermoreversibility in the range of 8–12 °C. Viscosity is directly proportional to the solution concentration, allowing the hydrogel parameters to be tailored for specific tasks. However, all measurements were performed in steady shear mode, and oscillatory tests are required for a full characterization of the viscoelastic properties. Due to the technical limitations of the available equipment (CP-4000 rotational viscometer), the storage (G′) and loss (G″) moduli, as well as the gelation point in oscillatory mode, were not determined. This limits the completeness of the rheological characterization of the material. However, this direction is planned in our further studies.
5. hBG gels possess a Young’s modulus of 4.67–4.89 kPa and a Bloom gel strength of 44.7 g. These parameters are lower than those of a commercial high-molecular-weight gelatin (CDH), characterizing hBG as a soft matrix. The low stiffness of hBG gels could potentially reduce shear stress during extrusion, which is hypothetically favorable for cells. However, this assumption requires experimental verification under conditions approximating bioprinting, including the assessment of post-printing shape fidelity and cell viability in printed constructs.
6. In vitro studies on human chondroblast spheroids confirmed the absence of hBG cytotoxicity. While chondroblast spheroids are a convenient and reproducible 3D model, they do not fully reflect the complexity of the bone tissue microenvironment. Cells maintain viability, morphology, and proliferative capacity both on the hydrogel surface and upon encapsulation within its structure (at concentrations of 5% and 10%). The retained collagen polypeptide chain promotes cell adhesion and the formation of the cell’s own extracellular matrix. These in vitro results should be interpreted with caution given the descriptive statistical approach (*n* = 3).

We also acknowledge that the presented rheological data are preliminary and do not allow definitive conclusions on the suitability of hBG as a bioink. This task requires a separate study employing oscillatory rheometry and thixotropy tests.

## 5. Conclusions

Thus, the developed method enables the production of highly purified gelatin from human bone tissue with stable physicochemical parameters. The obtained gelatin samples demonstrate a favorable soft-tissue stiffness profile, promoting long-term spheroid survival, which makes them a potential candidate biomaterial for use in 3D bioprinting. The relatively low viscosity/strength may be viewed as a potential advantage for reducing shear stress during extrusion; however, this assumption requires validation under real printing conditions using oscillatory rheological tests and assessment of post-printing cell viability. Extended oscillatory rheometry and thixotropy tests will be a priority in the next stage of work using a specialized rheometer.

The studied gelatin samples retain collagen polypeptide chains, which is important for cells, potentially enabling them to build their own extracellular matrix more rapidly.

To confirm the hypothesis of the retention of specific non-collagenous bone proteins (osteocalcin and osteopontin) within hBG and to assess their potential osteoinductive activity, an Enzyme-Linked Immunosorbent Assay (ELISA) and Western blotting using specific antibodies are planned for the next stage of the work.

For future applications in regenerative medicine, stabilization (crosslinking) of the material is necessary, with subsequent assessment of biodegradability by collagenase. The investigated hBG gelatin is non-crosslinked and water-soluble. At physiological temperature (37 °C), it dissolves rapidly in an aqueous environment without the need for enzymatic action. This limits its direct application as a long-term scaffold and necessitates the development of stabilization strategies. This stage is also planned for our subsequent studies.

A comprehensive mechanical characterization of crosslinked hBG derivatives, including relaxation tests and mathematical modeling of strength profiles, is planned as part of further studies. In the present work, we intentionally limited ourselves to basic static tests for non-crosslinked gelatin, as their results are sufficient to demonstrate the fundamental suitability of the material and do not mislead regarding the behavior of crosslinked forms, which will possess fundamentally different properties.

We plan to conduct experiments involving osteogenic differentiation of cells, as well as the use of co-culture models (e.g., chondroblasts with osteoblasts or endothelial cells). This will ultimately provide a more complete understanding of the developed material’s potential in bone tissue engineering. For future applications in regenerative medicine, stabilization (crosslinking) of the material is necessary, e.g., by methacrylation to obtain GelMA, with subsequent assessment of biodegradation and mechanical properties under physiological conditions. The studied hBG gelatin is non-crosslinked and water-soluble; at 37 °C it dissolves rapidly without enzymatic action, limiting its direct use as a long-term matrix.

Thus, at this stage hBG should be regarded more as a promising precursor and an object for further research aimed at optimizing its rheological properties and stabilization, rather than as a ready-to-use bioink. A final conclusion on its suitability for 3D bioprinting can only be drawn after a complete set of dynamic rheological tests and bioprinting experiments.

To assess the feasibility of using hBG in the engineering of human supporting and connective tissues, studies in animal models (e.g., an osteochondral defect model in rabbits) are also planned to evaluate the biocompatibility, osseointegration, and regenerative potential of stabilized hBG hydrogels in vivo. Prior to any in vivo application or clinical translation, mandatory testing for endotoxins (Limulus Amebocyte Lysate (LAL) test) and bioburden in accordance with regulatory requirements (e.g., ISO 10993-11 [48]) will be conducted. We plan to perform these tests in the next stage of material development.

## Figures and Tables

**Table 1:** Growth parameters of spheroids on gelatin of different concentrations.

Group	Day	Proliferation Index, Rel. Units	Doubling Time, h	Number of Doublings over 3 Days
0.1% (Control, BioloT LLC)	2	2.1	22.8	--
	3	1.7	31.0	1.8
10% (hBG-10)	2	2.0	24.0	--
	3	2.1	22.4	2.1
5% (hBG-5)	2	1.9	25.9	--
	3	2.1	22.4	2.0

## Data Availability

The original contributions presented in this study are included in the article. Further inquiries can be directed to the corresponding author.
